# Overexpression of *AtDREB1A* Causes a Severe Dwarf Phenotype by Decreasing Endogenous Gibberellin Levels in Soybean [*Glycine max* (L.) Merr.]

**DOI:** 10.1371/journal.pone.0045568

**Published:** 2012-09-18

**Authors:** Haicui Suo, Qibin Ma, Kaixin Ye, Cunyi Yang, Yujuan Tang, Juan Hao, Zhanyuan J. Zhang, Mingluan Chen, Yuqi Feng, Hai Nian

**Affiliations:** 1 The Guangdong Subcenter of National Center for Soybean Improvement, State Key Laboratory of Agricultural and Biological Resources Protection and Utilization in Subtropics, College of Agriculture, South China Agricultural University, Guangzhou, Guangdong, P. R. China; 2 Plant Transformation Core Facility, University of Missouri, Columbia, Missouri, United States of America; 3 College of Chemistry and Molecular Sciences, Wuhan University, Wuhan, Hubei, P. R. China; Instituto de Biología Molecular y Celular de Plantas, Spain

## Abstract

Gibberellic acids (GAs) are plant hormones that play fundamental roles in plant growth and developmental processes. Previous studies have demonstrated that three key enzymes of GA20ox, GA3ox, and GA2ox are involved in GA biosynthesis. In this study, the *Arabidopsis DREB1A* gene driven by the CaMV 35S promoter was introduced into soybean plants by *Agrobacterium*- mediated transformation. The results showed that the transgenic soybean plants exhibited a typical phenotype of GA-deficient mutants, such as severe dwarfism, small and dark-green leaves, and late flowering compared to those of the non-transgenic plants. The dwarfism phenotype was rescued by the application of exogenous GA_3_ once a week for three weeks with the concentrations of 144 µM or three times in one week with the concentrations of 60 µM. Quantitative RT-PCR analysis revealed that the transcription levels of the GA synthase genes were higher in the transgenic soybean plants than those in controls, whereas GA-deactivated genes except *GmGA2ox4* showed lower levels of expression. The transcript level of *GmGA2ox4* encoding the only deactivation enzyme using C_20_-GAs as the substrates in soybean was dramatically enhanced in transgenic plants compared to that of wide type. Furthermore, the contents of endogenous bioactive GAs were significantly decreased in transgenic plants than those of wide type. The results suggested that *AtDREB1A* could cause dwarfism mediated by GA biosynthesis pathway in soybean.

## Introduction

Gibberellic acids (GAs) are a class of essential hormones that play a key role in plant growth and developmental processes during the entire life cycle [1]. Three major oxidase gene families of *GA 20ox*, *GA3ox* and *GA2ox* participate in GA synthesis by a series of conversions from geranylgeranyl diphosphate [2]. The levels of GAs are homeostatically modulated through the negative feedback regulation of the expression of *GA20ox* and *GA3ox* genes and positive feed forward regulation of *GA2ox* genes [3], [4].

To date, there are eight *GA20ox* genes from *GmGA20ox1* to *GmGA20ox8*, six *GA3ox* genes from *GmGA3ox1* to *GmGA3ox*6, and ten *GA2ox* genes from *GmGA2ox1* to *GmGA2ox10* were identified in soybean, which were divided to four distinct subgroups (I, II, III, and C_20_ GA2ox) [5]. The *GA20ox* and *GA3ox* genes belong to subgroups I and II, respectively. The *GmGA2ox* genes except *GmGA2ox4* belong to subgroup III, which also includes *Arabidopsis GA2ox1* to *GA2ox6* [5]. The function of the subgroup III members are to deactivate bioactive GAs and hydroxylate C_19_-GA substrates [6]. In *Arabidopsis*, overexpression of *AtGA2ox1, -2, -3, -4, -5* and *-6* resulted in dwarfism and reductions in bioactive GA levels [6]. In contrast, knockout mutants of five C_19_-GA 2-oxidases genes showed lower bioactive GAs content and growth retardation, indicating that the *Arabidopsis* C_19_-GA 2-oxidases mainly inactivate GA pathway [6]. In soybean, *GmGA2ox4* may potentially receives only C_20_ (GA_12_ and GA_53_, precursors of bioactive GAs) as substrates and belongs to subgroup C_20_ GA2oxs [5], which also includes *AtGA2ox7* and *AtGA2ox8*, *spinach GA2ox3*, and *OsGA2ox4, -5, -6.* Ectopic expression of *AtGA2ox7* and *AtGA2ox8* in transgenic *tobacco* (*Nicotiana tabacum*) also led to a dwarf phenotype [7]. This was also found with the activation of *OsGA2ox6* in rice [8]. However, C_20_ GA2oxs were found to cause less severe GA-defective phenotypes than C_19_ GA2oxs in rice [9].

DREB (dehydration responsive element binding) transcription factors encode dehydration responsive element binding protein (DREB1 and DREB2) and contain a conserved AP2/EREBP motif. DREB specifically interacts with the dehydration-responsive element/C-repeat (DRE/CRT) *cis*-acting element, triggers the expression of downstream stress-related genes and confers plants improved tolerance to drought, low temperature and high salinity [10], [11]. Interestingly, overexpression of *CBF3/DREB1A* and other *DREB1s* members under the control of the CaMV 35S promoter caused severe retardant growth of plants including *Arabidopsis* [12]–[16], *tobacco* [17]–[19], and *chrysanthemum* [20]. Exogenous GA_3_ treatment reversed the dwarfism caused by overexpression of *DREB1B* and *DREB1F* [14], [16], [17], but failed to rescue the dwarfism by overexpression of *AtDREB1A* in *Arabidopsis* and *tobacco* [15], [18], [19].

Here, we reported that overexpression of *AtDREB1A* in soybean plants caused dwarf phenotype, which can be rescued by the application of exogenous GA_3_. The transcript expression level of *GmGA2ox4* was up-regulated in transgenic soybean plants, which decreased the levels of bioactive GAs as regarding on the dwarfism of soybean.

## Materials and Methods

### Plasmid Construction

The plasmids pUC18 (TaKaRa) deleted the sites between *Bam*HI and *Pst*I and the paragraph of pZY102 with 35S-GUS-NOS sequence were digested with restriction endonuclease *Hin*dIII, and then the two linearized parts were linked together (thereafter named as pUC18-pZY102). A 663 bp opening reading frame (ORF) of *AtDREB1A* was amplified from the cDNA of *Arabidopsis* ecotype *Columbia* using reverse transcriptase PCR and ligated into pGEM -T Easy vector at the multiple cloning site (Promega). The primers were designed as 5′GGATCCTTTCAGCAAACCATACCA3′ and 5′GGTACCCACTCGTTTCTCGTTTTA3′ with the *Bam*HI and *Kpn*I sites, respectively. The ORF paragraph of *AtDREB1A* digested with *Bam*HI/*Kpn*I was cloned into the site of GUS position of the intermediate vector of pUC18-pZY102. After sequencing confirmation, the paragraph of 35S-*AtDREB1A*-NOS from pUC18- pZY102 was inserted into pZY101 vector at *Hin*dIII site, which was named pZY101-*AtDREB1A*. The resulting binary vector was introduced into *Agrobacterium tumefaciens* strain EHA101 by the freeze-thaw method [21], which was then used for further genetic soybean transformation.

### Soybean Transformation

Mature soybean seeds of cultivar Huachun 5 bred in Guangdong Subcenter of National Center for Soybean Improvement were surface sterilized for 13.5 h using chlorine gas produced by mixing 4.2 ml of 12 N HCl with 100 ml sodium hypochlorite in tightly sealed desiccators [22]. The cotyledonary-node method described herein was modified from that described previously [23] and the brief methodology is given below.

Each of explants was prepared by removing the root and the majority of the hypocotyl approximately 3–5 mm below the cotyledonary-node after four days germination in germination medium(B5 salt/B5 vitamins, 30 g/L sucrose, 3 g/L phytagel, pH 5.8). The cotyledonary-nodes were wounded by making 10 slices with the blade perpendicular to the hypocotyls and inoculated in the 30 ml co-cultivation suspension for 30 min, and then transferred on co-cultivation medium (B5 salt (0.1x)/B5 vitamins, 30 g/L sucrose, 3 g/L phytagel, 3.9 g/L MES, 0.25 mg/L GA_3_, 0.15 g/L Na-thiofate, 0.4 g/L L-cysteine, 0.15 g/L DL-dithiothreitol, 0.04 g/L Acetosyringone, pH 5.4) as abaxial side down under dark condition. Three days later, the infected explants were briefly washed in washing medium, and transferred to shoot inducing medium(B5 salt/B5 vitamins, 30 g/L sucrose, 3 g/L phytagel, 0.59 g/L MES,1.67 mg/L 6-BA, 100 mg/L Timentin, 200 mg/L Cefotaxime, 5 mg/L Glufosinate, pH 5.7) and shoot elongation medium (MS salt/MS vitamins, 30 g/L sucrose, 3 g/L phytagel, 0.59 g/L MES, 5 mg/L Asparagine, 5 mg/L Glutamine, 0.4 mg/L IAA, 0.5 mg/L GA_3_, 1 mg/L Trans-Zeatin Riboside, 100 g/L Timentin, 200 mg/L Cefotaxime, 5 mg/L Glufosinate, pH 5.7), cultured for four weeks respectively. Elongated shoots were placed into rooting medium containing 0.5 mg/L IBA. Primary positive plants were screened with 135 mg/L Liberty (AgrEvo) [24], and identified by DNA and RNA analysis.

### Exogenous GA_3_ Treatment

Three-week-old transgenic soybean seedlings of T_3_ generation were sprayed with a GA_3_ solution of 0, 60, 144, or 288 µM (in 10% ethanol) once a week for three consecutive weeks or with a GA_3_ solution of 60 µM (in 10% ethanol) three times in one week. The plants of wide type were treated with 10% ethanol as control. The plant height, leaf area and chlorophyll content were measured two weeks after the treatment.

### Chlorophyll Content Measurement

The chlorophyll content of the first and second expanding trifoliates in transgenic and wide-type plants were measured by Portable chlorophyll content meter (SPAD-502, Spectrum Technologies, Inc), and each measurement repeated three times.

### Gene Expression Analysis

The six-week-old soybean seedlings of transgenic and wide type were extracted using Trizol reagent (Invitrogen). After RNase-free DNase (TaKaRa) treatment, approximately 1 µg total RNA was used for reverse transcription using the oligo (dT) primer and M-MLV (Invitrogen). qRT-PCR was performed using CFX96 (Bio-Rad, USA) and SYBR Green I (Bio-Rad, USA). Each of the cDNA samples was subjected to a real-time PCR analysis in triplicate. The data were normalized using the reference gene *β-tubulin*. The relative expressions of specific genes were quantified using the 2^–ΔΔCt^ calculation. The primer pairs used for q- RT-PCR are listed in Table S1.

### Quantification of Endogenous GAs

The transgenic and wide type soybean plants were grown in 1/2 Hoagland solution for four weeks in growth chamber under 28°C, 16-h light and 24°C, 8-h dark condition. Samples were taken from the top part of young plants including apex, young stem and young leaves. The GAs contents were determined by the method of capillary electrophoresis-time of flight-mass spectrometry described previously [25].

## Results

### Overexpression of the *Arabidopsis DREB1A* Gene in Soybean Caused Severe Dwarf Phenotype

**Figure 1:**
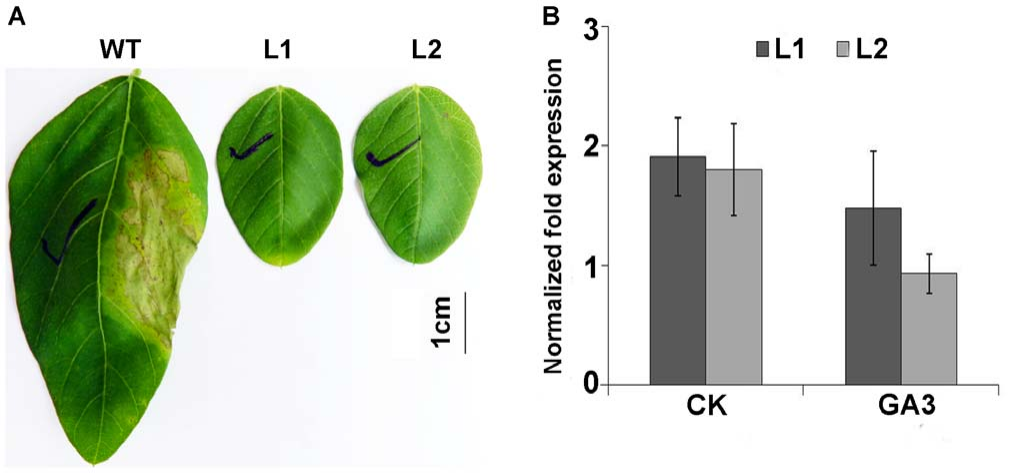
Herbicide tolerance and qRT-PCR analysis of transgenic plants. A: Detection of herbicide tolerance on *AtDREB1A-*transformed plants. B: Expression analysis of the *AtDREB1A* gene in transgenic plants. Data represents six biological replications, and error bars represent SE. WT, wide type; L1: *AtDREB1A* transgenic line 1; L2: *AtDREB1A* transgenic line 2; CK: control check, without GA_3_ treatment; GA_3_: 144 µM GA_3_ treatment.

The *Arabidopsis DREB1A* gene driven by the CaMV 35S promoter was transferred into the soybean plants using *Agrobacterium*-mediated transformation of the cotyledon node. Unexpectedly, during the transformation process, some elongated shoots showed abnormal phenotype with no obvious stems (Fig. S1). Consequently, these shoots were later identified as positive plants. A total of 12 T_0_ lines were successfully regenerated. All the *35S::AtDREB1A* transgenic plants exhibited severe dwarf phenotype (Fig. S2). Homozygous T_3_ plants of two independent transgenic lines of *AtDREB1A*-L1 and *AtDREB1A*-L2 were selected for further analysis. The transgenic lines were more tolerance to the herbicide treatment than that of wide type (Fig. 1A). Moreover, qRT-PCR analysis showed the *AtDREB1A* was transcriptionally expressed in transgenic lines. However, under the GA_3_ treatment condition, its expression was decreased (Fig. 1B).

**Figure 2:**
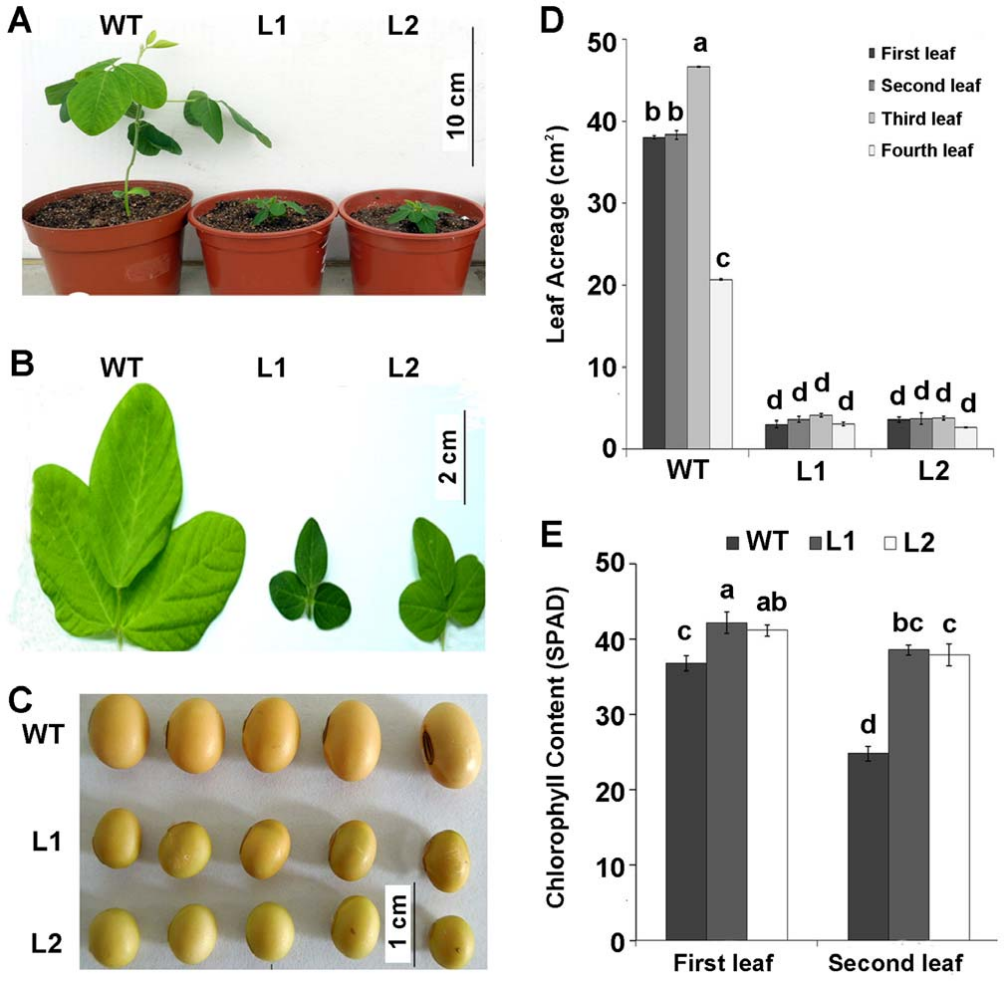
Phenotypes of *AtDREB1A*-overexpressing soybean plants. A: Phenotypes of 3-week-old T_3_ transgenic and WT soybean plants. B: Leaf phenotype of 3-week-old T_3_ transgenic and WT soybean plants. C: Seeds of transgenic and WT soybean plants. D: The first to fourth leaf acreage of 3-week-old transgenic and WT soybean plants. E: The chlorophyll content of first and second leaf in transgenic and WT soybean plants. WT: wide type; L1: *AtDREB1A* transgenic line 1; L2: *AtDREB1A* transgenic line 2. Values are the mean of six biological replicates ± SE, the same letter on each column set indicates no significant difference and different letters are significantly different by the analysis of variance (ANOVA), p<0.05.

The *35S::AtDREB1A* transgenic plants exhibited a severe dwarf phenotype with no observable internodes (Fig. 2A). The average length of internodes was only 19.58% and 22.08% of those in wide type, respectively. The height of transgenic plants was decreased by 79.91% and 80.05% of those in wide type, respectively (Table 1). The leaf area and color from the 1^st^ trifoliate to 4^th^ trifoliate were smaller and darker than those of wide-type (Fig. 2B, 2D, 2E). The chlorophyll contents of the 1^st^ and 2^nd^ trifoliate were 1.1-fold and 1.5-fold higher in transgenic plants than those of control plants (Fig. 2E). In addition, the transgenic plants showed the phenotypes of late flowering and podding. The flowering and podding stage were longer more than 20 days and 40 days than those of wide type, respectively (Table 1, Fig. S3). Furthermore, the transgenic seeds were much smaller in size with the grain weight only about 51.5% and 55.2% of those of wild type (Fig. 2C, Table 1).

**Table 1:** Characterization of transgenic and WT soybean plants.

Traits	WT	L1	L2
Plant height (cm)	35.00±2.36A	7.03±0.58B	6.98±0.34B
Average length of Internodes (cm)	2.40±0.20A	0.47±0.03B	0.53±0.01B
Initial flower stage (day)	29.00±1.84A	52.00±2.06B	53.00±1.72B
Initial pod stage (day)	44.00±1.12A	90.00±1.68B	91.00±1.02B
Grain weight(g/100 grains)	30.43±0.26A	8.63±0.20B	3.35±0.26B

Values are the mean of ten biological replicates ± SE, the same letter indicates no significant difference and different letters are significantly different by the analysis of variance (ANOVA), p<0.01. WT: wide type; L1: *AtDREB1A* transgenic line 1; L2: *AtDREB1A* transgenic line 2.

### The Dwarf Phenotype of Transgenic Soybean were Rescued by the Application of Exogenous GA_3_

**Figure 3:**
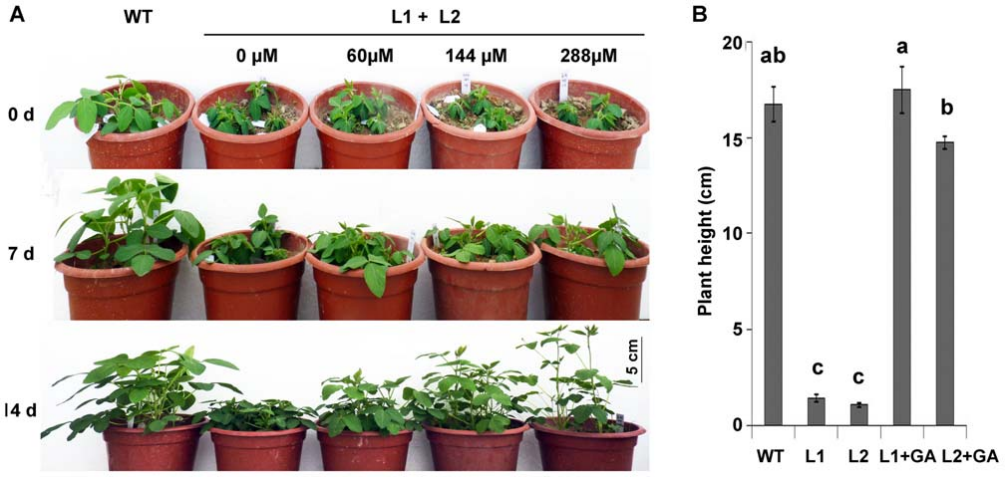
Effects of GA_3_ on phenotypic restoration. A: Phenotypes of transgenic plants under 0 µM, 60 µM, 144 µM or 288 µM GA_3_ treatments for 7 days and 14 days. B: The plant height of eight weeks of WT and transgenic plants after treated with or without 144 µM GA_3_ for two weeks. WT: wide type; L1: *AtDREB1A* transgenic line 1; L2: *AtDREB1A* transgenic line 2; L1+L2: *AtDREB1A* transgenic plants line1 and 2. Values are the mean of six biological replicates ± SE, the same letter on each column set indicates no significant difference and different letters are significantly different by the analysis of variance (ANOVA), p<0.05.

Overexpression of *AtDREB1A* caused dwarfism, dark-green leaves and late flowering, which resembles the previously identified typical phenotypes of GA-deficiency mutants [13]. This suggested that the phenotypic changes of *AtDREB1A-*overexpression plants were caused by GA_3_ deficiency. Application of GA_3_ at concentration of 144 µM once a week for three consecutive weeks rescued the dwarf phenotype in transgenic plants (Fig. 3). In addition, when application of GA_3_ at concentration of 60 µM and increased frequency to three times in one week, the plant height of transgenic plants also be rescued and even much taller than that of plants under 144 µM GA_3_ treatment and wide type (Fig. S4). The leaf area of was partly rescued and chlorophyll contents was fully rescued after GA_3_ treatment in transgenic plants (data not shown). However, the flowering time was didn’t rescued (data not shown).

### The Expression of GA Biosyntheses Genes were Changed in the Overexpression of *AtDREB1A* Soybean Plants

**Figure 4:**
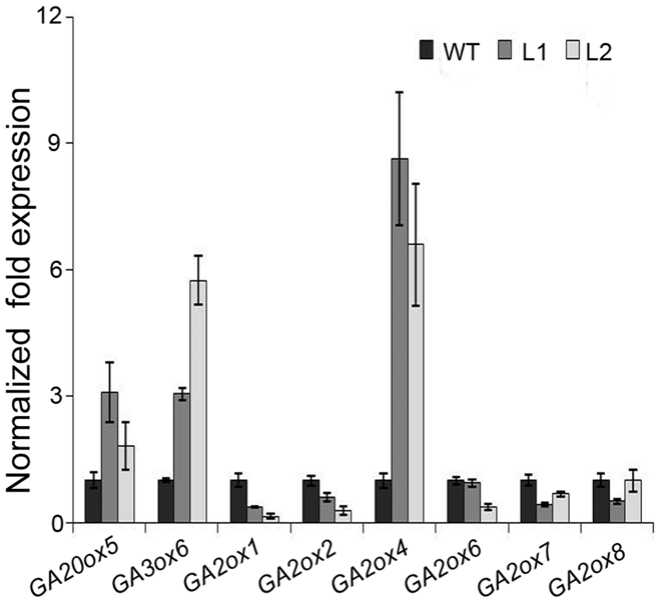
Expression levels of GA metabolism related genes. *β*-*Tubulin* was used as an internal control. *GA20ox5*: *GmGA20ox5* (Glyma13g09460); *GA3ox6*: *GmGA3ox6* (Glyma17g30800); *GA2ox1*: *GmGA2ox1* (Glyma02g01330); *GA2ox2*: *GmGA2ox2* (Glyma10g01380); *GA2ox*4: *GmGA2ox4* (Glyma11g00550); *GA2ox6*: *GmGA2ox6* (Glyma13g33290); *GA2ox7*: *GmGA2ox7* (Glyma13g33300); *GA2ox8*: *GmGA2ox8* (Glyma15g10070). Values are the mean of four biological replicates ± SE.

The GA-20 oxidase, GA-3-β-hydroxylase and GA-2 oxidase are critical enzymes in GA biosyntheses. Quantitative RT-PCR analysis was performed to investigate these genes expression level in transgenic plants. Due to the tissue specific expression pattern among members, only *GmGA20ox5*, *GmGA3ox6* and six GA2- oxidase genes were detected. The result showed that the relative mRNA expression of *GmGA20ox5* and *GmGA3ox6* were dramatically increased in two transgenic lines compared with those of wild-type plants (Fig. 4). While the mRNA level of GA2-oxidase genes of *GmGA2ox1, GmGA2ox2, GmGA2ox6, GmGA2ox7* and *GmGA2ox8* were down regulated in transgenic lines (Fig. 4). However, the transcriptional expression of another GA2-oxidase gene, *GmGA2ox4*, was significantly up-regulated in transgenic plants compared to that of wide type (Fig. 4).

### Overexpression of *AtDREB1A* Reduced the Bioactive GAs

Regarding the data above, we propose that the GA biosynthesis was interfered by overexpression of *AtDREB1A* through regulating the GA synthase genes, especially for activating the expression of *GmGA2ox4,* which could decrease the bioactive level in transgenic soybean plants. Thus, the endogenous GAs content were examined using collected samples of apex, young leaves and stem of transgenic and wide type soybean seedlings after hydroponic cultivation for four weeks. Table 2 showed that the concentration of the bioactive GA_1_ in transgenic plants was reduced by 74.41% compared with that of wide type, and the concentration of bioactive GA_4_ was even not detected. It demonstrated that *AtDREB1A* transgenic plants are similar to GA deficient mutants. The contents of intermediates (GA_19_) and producers (GA_9_ and GA_20_) of GA20ox were decreased to 13.94%, 28.01% and 24.58% of wide type in transgenic plants, respectively. Based on this observation, we speculated that GA20-oxidation might be partly impaired in transgenic plants, which was consistent with previous report [14]. Furthermore, some C_20_-GAs (GA_12_, GA_53_ and GA_24_) in transgenic plants accumulated at lower levels below the limit of detection compared with those of wide type. Taken together, these results indicate that the deficiency of bioactive GAs in transgenic soybean plants is mainly because of the inhibition of stepwise oxidation catalyzed by C_20_-GA2ox.

**Table 2:** The bioactive GAs contents in transgenic and WT plants.

GAs	WT	L1
Non-13-hydroxylated		
GA12	0.39±0.08	ND
GA24	1.39±0.17	ND
GA9	7.10±0.29	0.99±0.12
GA4	5.00±0.15	ND
13-Hydroxylated		
GA53	0.61±0.10	ND
GA19	5.64±0.23	1.58±0.19
GA20	4.12±0.48	0.24±0.08
GA1	6.55±0.33	1.61±0.14

3-week-old soybean seedlings of transgenic and wide type were sampled for GA content measurement. WT: wide type; L1: *AtDREB1A* transgenic line 1. All values are ng/g fresh weight, data represents four biological replicates. ND, not detectable.

## Discussion

In this study, we demonstrated that overexpression *AtDREB1A* in soybean caused a dwarfism phenotype, probably by the up-regulation of the *GmGA2ox4* gene, which resulted in decreasing levels of active GAs and conferred dwarfism phenotypes. Overexpression of *AtDREB1A* transcription factor in soybean caused dwarfism (Fig. 2, Table 1). Similar phenomena was also found when DREB members were overexpressed in other plant species, such as *Arabidopsis* [12], [13], *tobacco* [18], [19] and *chrysanthemum* [20]. The dwarfism caused by overexpression of *DREB1B* and *DREB1F* in *Arabidopsis* can be reversed by exogenous GA_3_ treatment [14], [16], [17]. However, the dwarfism caused by overexpression of *DREB1A* in *Arabidopsis* and *tobacco* cannot be reversed by GA treatment [15], [18], [19]. In this research, exogenous GA_3_ treatment restored the plant height (Fig. 3, Fig. S4). This suggested that the different DREB-like transcription factors or the same DREB transcription factors but in different transgenic plant backgrounds may contribute to plant growth differentially [26].

We detected the expression of GA-20-oxidase and GA-3-oxidase genes which involve in GA biosyntheses and GA2-oxidase which convert bioactive GAs into deactivated forms [27]. In contrast, the transcripts of *GmGA20ox5* and *GmGA3ox6* were both up-regulated in transgenic soybean plants compared with that in wide type (Fig. 4). It seems that the inhibition of GA biosynthesis does not account for the transcriptional repression of GA-20-oxidase or GA-3- oxidase genes. Similarly, previous studies found that the expression of *AtGA20ox1*, *AtGA20ox2*, *AtGA20ox3* and *AtGA3ox1* were up-regulated in *35S::DREB1F* dwarf plants [14]. Meanwhile, the expression of *Gh20ox1-4*, *Gh3ox1* and *Gh3ox2* were also increased in *35S::GhDREB1 Arabidopsis* [28]. In addition, the up-regulation of GA-20-oxidase and GA-3-oxidase genes has been reported in GA-deficient and GA-insensitive mutants [4], [29]. These suggested that the up-regulation of *GmGA20ox5* and *GmGA3ox6* in *35S::DREB1A* soybean plants may due to the negative feedback regulation of endogenous GAs levels. However, the transcriptional expression of one GA deactivating gene of *GmGA2ox4* was up-regulated, while the transcriptional expression of other GA deactivating genes were down-regulated compared to those of wide type (Fig. 4). Previous study predicted that *GmGA2ox4* only hydroxylates C_20_-GA rather than the C_19_-GA substrates in soybean, which is clustered into the same subgroup of C_20_ GA2ox with *spinach GA2ox3*, *AtGA2ox7, AtGA2ox8*, and *OsGA2ox4*,-5,-6 [5], [27]. Amino acid sequence alignment showed that GmGA2ox4 was closed to AtGA2ox7 and AtGA2ox8 than any other GA-2 oxidase in soybean and has the conserved motifs for binding GAs and other common cofactors (Fig. S5). However, a unique region (at the positions 115 to 143 of AtGA2ox8) in C_20_-GA subgroup may define the specificity of the reactions performed by these enzymes. It has been reported that overexpression of *AtGA2ox7* and *AtGA2ox8* decreased the levels of active GAs and conferred dwarf phenotypes both in *Arabidopsis* and *tobacco* [7]. Consistent with this observation, homologous and heterogonous over-expression of rice *GA2ox5* and *GA2ox6* resulted in typical GA-deficient dwarfism [9]. Similarly, transgenic tobacco of overexpression of *spinach GA2ox3* showed dwarf phenotype [27]. In this research, overexpression of *AtDREB1A* in soybean increased expression level of *GmGA2ox4,* resulted in typical GA-deficient dwarfism and decreased the active GAs levels (GA_1_ and GA_4_). What’s more, the bioactive levels of C_20_-GAs (GA_12_, GA_53_ and GA_24_) in transgenic plants were lower than limit of detection (Table 2). Recently reports showed that *GA2ox7* and *GA2ox8* in *Arabidopsis* and *GA2ox3* in *spinach* hydroxylate C_20_-GA precursors (GA_12_ and GA_53_) [7], [15], [27]. In addition, GA_24_ could metabolize by GA2ox7 in *vitro,* suggesting GA_24_ is another substrate of GA2ox7 [15]. Therefore, we tentatively propose that GA_12_, GA_53_ and GA_24_ were substrates of GmGA2ox4 in soybean and C_20_-GA oxidation plays an important role in resulting GA deficiency in transgenic soybean plants.

Overexpression of *AtDREB1A* modulates plant growth through regulating C_20_-GA deactivation genes with a similar mechanism as found in other plants. In *35S::DDF1* transgenic plants, the expression of *AtGA2ox7* were dramatically increased (223-fold) compared to control plants [15]. DDF1 protein can bind to DRE-L motifs in the *GA20x7* promoter, suggesting that *GA20x7* is a direct target of DDF1 transcriptional activator [15]. In addition, the expression of *GA2ox3* was up-regulated in CBF1 overexpression plant while there is no CRT/DRE-like *cis*-element in the promoter region of *GA2ox3*, implying that CBF1 up-regulated *GA2ox3* gene expression indirectly [16]. In this study, no CRT/DRE-like *cis*-element was found in the promoter region of *GmGA2ox4*. Alternatively, there exist two ERE (ethylene-responsive element) elements with a core sequence of AGCCGCC, and some DREBs such as TINY2, BnDREBIII-1 and CBF1/DREB1B were demonstrated to bind to ERE element [30]–[32]. However, CBF2/DREB1C and CBF3/DREB1A have been demonstrated without binding to ERE due to 15^th^-Cys other than Ser like in TINY, TINY2, BnDREBIII-1, which is crucial for the specific binding of ERE element [33]. Therefore, the results suggested that *AtDREB1A* regulates *GmGA2ox4* gene expression through an indirectly way.

It was widely reported that overexpressing DREBs in plants increased the transgenic plants tolerance to abiotic stresses [12]–[20]. It was reported that soybean lines transformed with an *rd29A::AtDREB1A* construct improved the tolerance to drought [34], suggesting that *AtDREB1A* is involved in stress tolerance in soybean. In this study we demonstrated that overexpression of *AtDREB1A* gene up-regulated the expression of the only C_20_-GA-oxidase *GmGA2ox4*, which decrease the active GAs and corresponding for dwarf phenotype in soybean.

Taken together, we showed that overexpression *AtDREB1A* in soybean could result in a typical phenotype of GA-deficient mutants including severe dwarfism, small and dark-green leaves, and late flowering in transgenic plants. The dwarfism phenotype could be rescued by the application of exogenous GA3 with the concentrations of 60 µM or 144 µM. The dwarfism of 35S::*AtDREB1A* reveals *AtDREB1A* can mediate GA metabolism and regulate some GA-responsive genes involved in the GA synthase genes and GA deactivated genes, which were further confirmed by the contents of endogenous bioactive GAs. The gained information suggested that *AtDREB1A* causes soybean dwarfism mediated by GA biosynthesis pathway.

## Supporting Information

Figure S1 **The phenotype of transgenic and wide-type shoots during the period of tissue culture.** A: wild type; B: regenerate shoots.
(TIF)

Figure S2 **The phenotypes of transgenic plants after transferred into pots.** A: wide type; B–H: transgenic plants.
(TIF)

Figure S3 **The phenotypes of transgenic plants during the growth and development.** A, E and I: wild type at vegetable stage, flowering and podding stage. C, G and K are the magnified pictures of wild type plants corresponding to A, E and I, respectively. B, F and J: *AtDREB1A* transgenic plants at vegetable, flowering and podding stage. D, H and L are the magnified pictures taken from the top of *AtDREB1A* transgenic plants corresponding to B, F and J, respectively.
(TIF)

Figure S4 **Effects of GA_3_ on phenotypic restoration.** A: Phenotypes of transgenic plants under 60 µM and 144 µM GA3 treatments. B: The plant height of transgenic and WT soybean plants after treated with or without GA3 treatment for two weeks. WT: wide type; L1: *AtDREB1A*-transgenic line 1; L2: *AtDREB1A* transgenic line 2; Values are the mean of six biological replicates ± SE, the same letter on each column set indicates no significant difference and different letters are significantly different by the analysis of variance (ANOVA), p<0.05.
(TIF)

Figure S5 **Sequence alignment of predicted proteins of C_20_-oxidase group.** Black shading indicates identical amino acid residues, and gray shading indicates similar residues. GenBank accession numbers of proteins are (in parentheses): AtGA2ox7 (At1g50960), AtGA2ox8 (At4g21200), OsGA2ox5 (Os07g01340), OsGA2ox6 (Os04g44150), SoGA2ox3 (AAX14674), OsGA2ox9 (Os02g41954), GmGA2ox4 (Glyma11g00550).
(TIF)

Table S1 **Primers used for real-time quantitative RT-PCR.**
(TIF)
