# Supplementary figures and images for: Overexpression of AtDREB1A Causes a Severe Dwarf Phenotype by Decreasing Endogenous Gibberellin Levels in Soybean [Glycine max (L.) Merr.]
Source: PLoS One. 2012 Sep 18;7(9):e45568. doi: 10.1371/journal.pone.0045568 (PMC3445508; doi:10.1371/journal.pone.0045568)

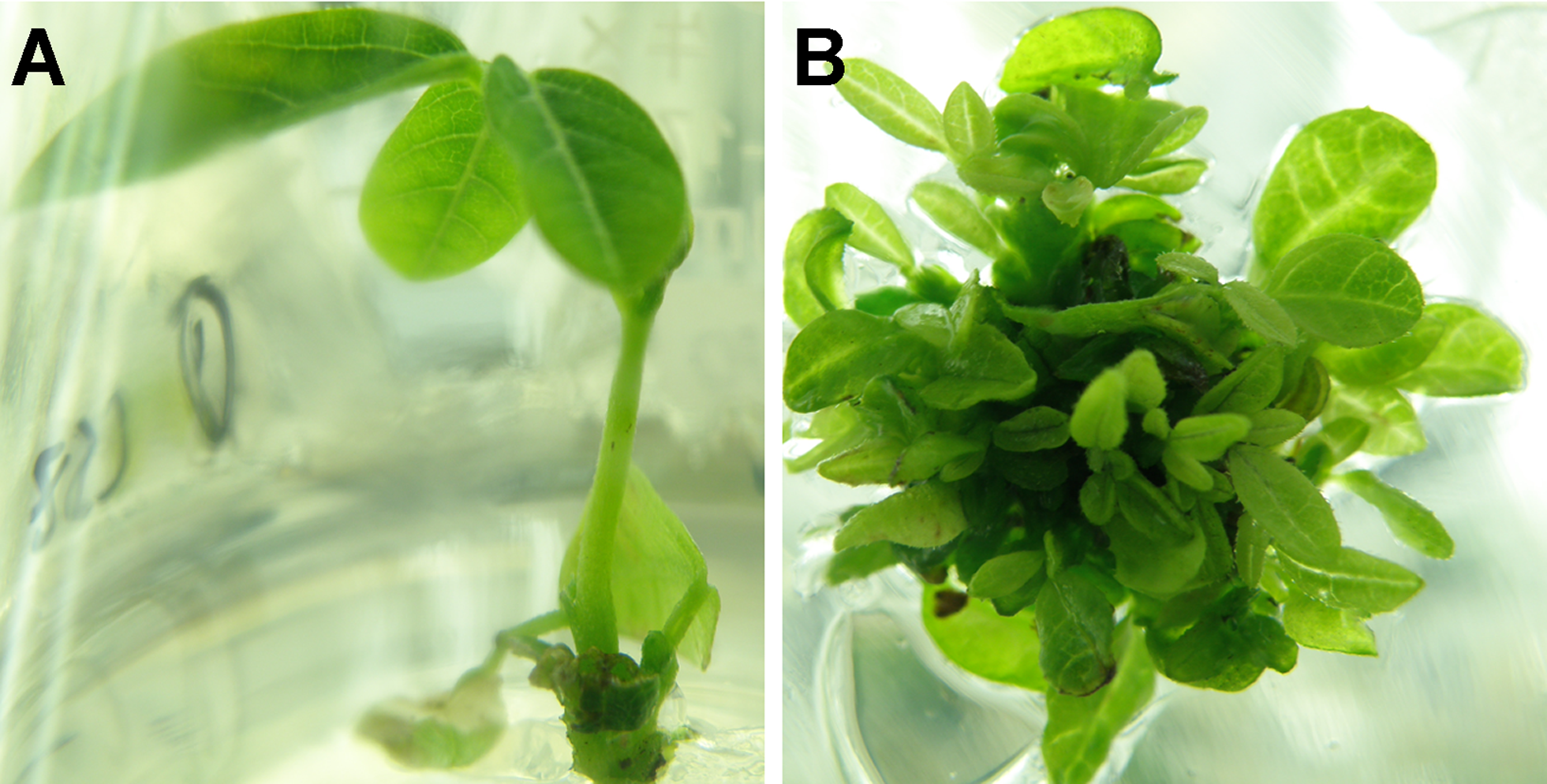

Supplement: Figure S1 — The phenotype of transgenic and wide-type shoots during the period of tissue culture. A: wild type; B: regenerate shoots. (TIF) [file pone.0045568.s001.tif]

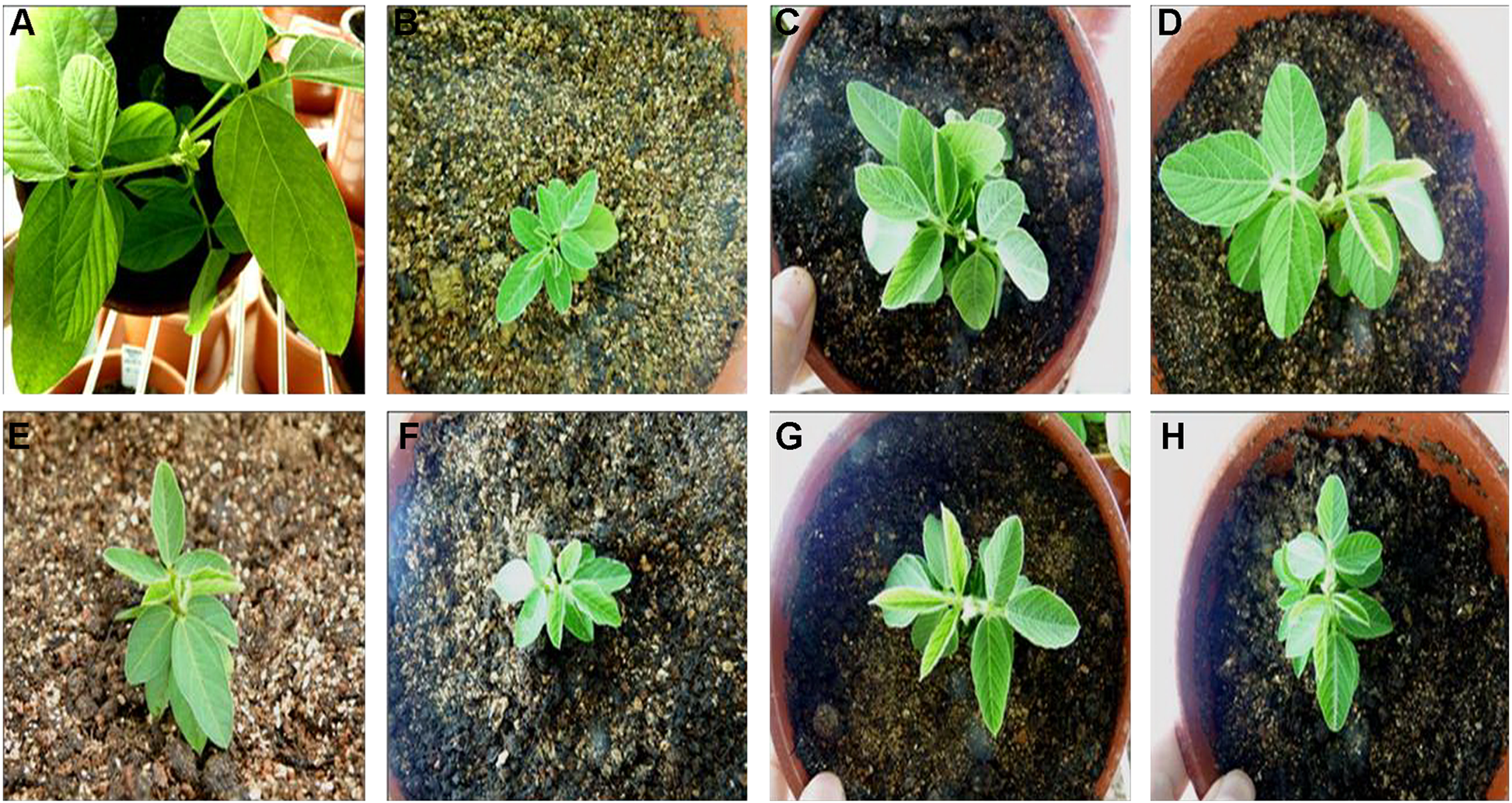

Supplement: Figure S2 — The phenotypes of transgenic plants after transferred into pots. A: wide type; B–H: transgenic plants. (TIF) [file pone.0045568.s002.tif]

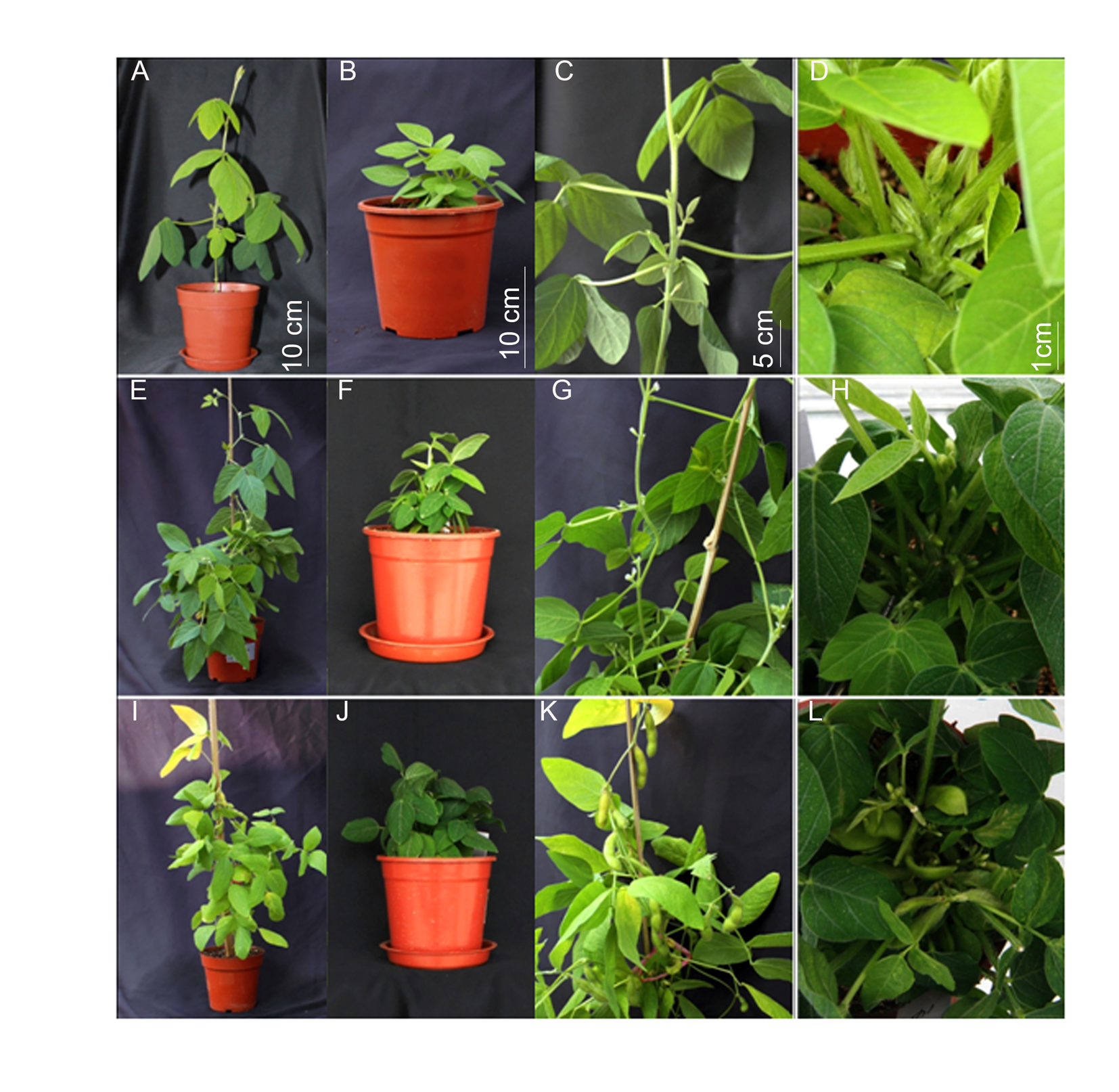

Supplement: Figure S3 — The phenotypes of transgenic plants during the growth and development. A, E and I: wild type at vegetable stage, flowering and podding stage. C, G and K are the magnified pictures of wild type plants corresponding to A, E and I, respectively. B, F and J: AtDREB1A transgenic plants at vegetable, flowering and podding stage. D, H and L are the magnified pictures taken from the top of AtDREB1A transgenic plants corresponding to B, F and J, respectively. (TIF) [file pone.0045568.s003.tif]

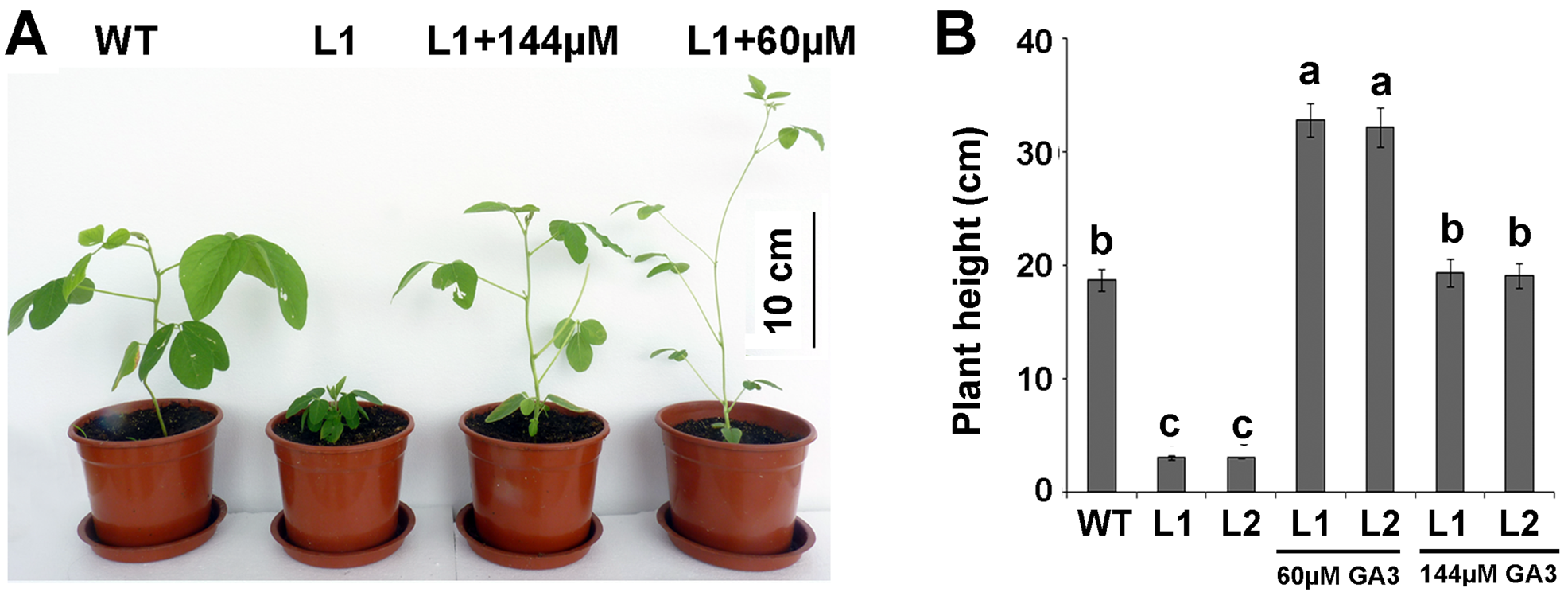

Supplement: Figure S4 — Effects of GA3 on phenotypic restoration. A: Phenotypes of transgenic plants under 60 µM and 144 µM GA3 treatments. B: The plant height of transgenic and WT soybean plants after treated with or without GA3 treatment for two weeks. WT: wide type; L1: AtDREB1A-transgenic line 1; L2: AtDREB1A transgenic line 2; Values are the mean of six biological replicates ± SE, the same letter on each column set indicates no significant difference and different letters are significantly different by the analysis of variance (ANOVA), p<0.05. (TIF) [file pone.0045568.s004.tif]

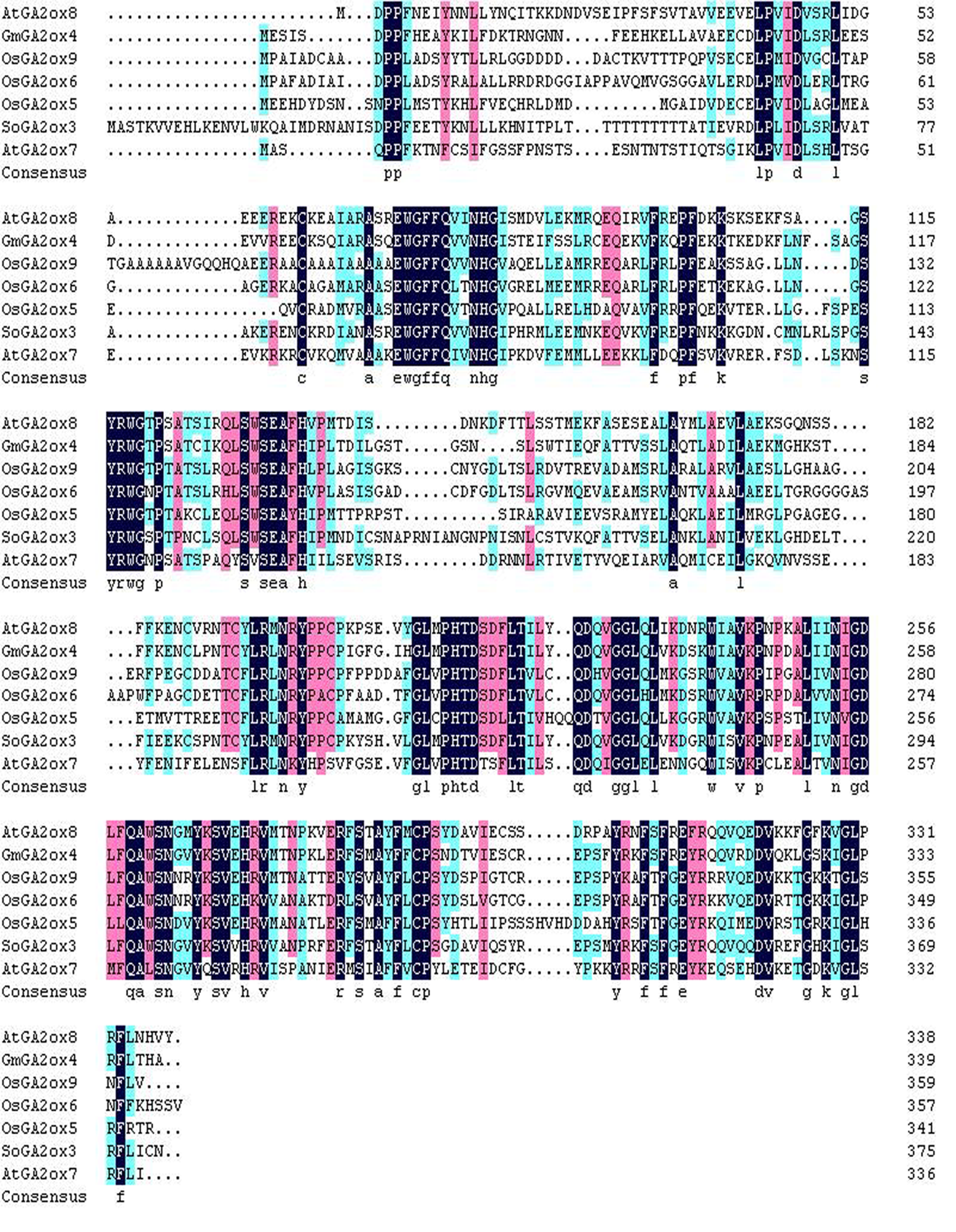

Supplement: Figure S5 — Sequence alignment of predicted proteins of C20-oxidase group. Black shading indicates identical amino acid residues, and gray shading indicates similar residues. GenBank accession numbers of proteins are (in parentheses): AtGA2ox7 (At1g50960), AtGA2ox8 (At4g21200), OsGA2ox5 (Os07g01340), OsGA2ox6 (Os04g44150), SoGA2ox3 (AAX14674), OsGA2ox9 (Os02g41954), GmGA2ox4 (Glyma11g00550). (TIF) [file pone.0045568.s005.tif]

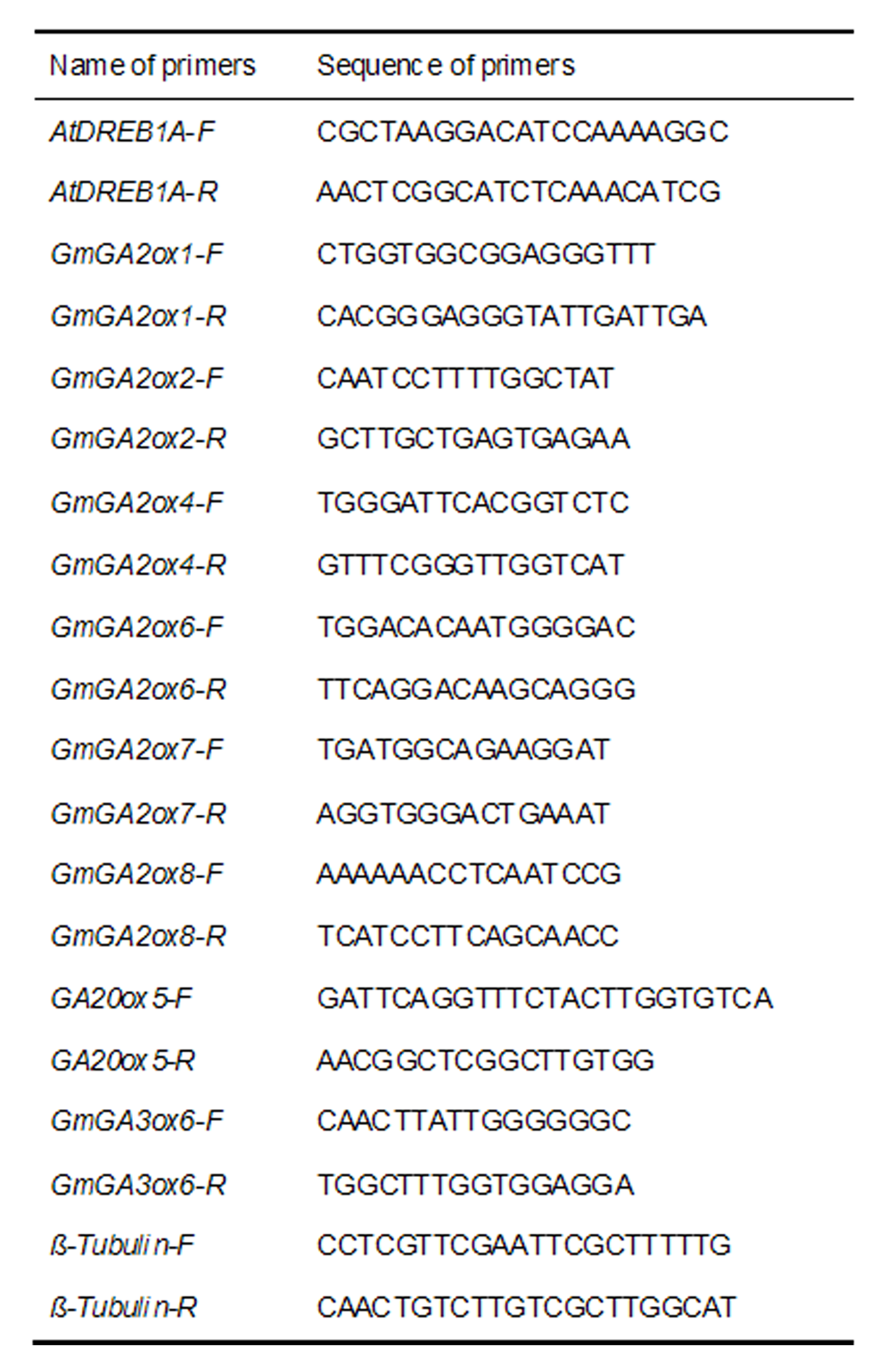

Supplement: Table S1 — Primers used for real-time quantitative RT-PCR. (TIF) [file pone.0045568.s006.tif]
